# Unusual cause of a painful right testicle in a 16-year-old man: a case report

**DOI:** 10.1186/1752-1947-5-27

**Published:** 2011-01-21

**Authors:** Khalid N Shehzad, Amjid A Riaz

**Affiliations:** 1 Department of Surgery, Watford General Hospital, Vicarage Road, Watford, West Hertfordshire WD18 0HB, UK

## Abstract

**Introduction:**

Urgent surgical exploration of the scrotum of a child or teenager who presents with a painful and swollen testicle is paramount if testicular torsion is not to be missed. It is extremely rare for a non-scrotal pathology to present with acute scrotal signs. Here we present such a rare case and emphasize the importance of being aware of this potential clinical pitfall.

**Case presentation:**

A 16-year-old Caucasian man presented as a surgical emergency with a five to six hour history of a painful, red, and swollen right hemiscrotum. He also complained of vague lower abdominal pain, vomiting, and watery diarrhea. He had a temperature of 38.5°C and a tender, red, and swollen right hemiscrotum. The right testicle appeared elevated. He was mildly tender in his central and upper abdomen and less so in the lower abdomen. No convincing localizing abdominal signs were noted. He had an increased white cell count (15 × 10^9^/L) and C-reactive protein (CRP; 300 mg/L). Urgent right hemiscrotal exploration revealed about 5 ml of pus in the tunica vaginalis and a normal testicle. A right iliac fossa incision identified the cause: a perforated retrocecal appendix. Appendectomy was performed, and both the abdomen and scrotum washed copiously with saline before closure. The patient made an uneventful recovery.

**Conclusion:**

Acute appendicitis presenting with scrotal signs due to a patent processus vaginalis is an extremely rare clinical entity. To date, fewer than five such cases have been reported in the medical literature. It is, therefore, extremely important to be aware of this unusual clinical scenario, as only a high index of suspicion will enable prompt, successful management of both the appendicitis and the scrotal abscess.

## Introduction

A painful, swollen testicle is a common surgical emergency, especially in young children and teenagers. The diagnosis that must be excluded is testicular torsion. Prompt surgical exploration of the scrotum is important, as delay can jeopardize the viability of a twisted testicle. It is very rare for a non-testicular pathology to present with acute scrotal signs. Such an unusual clinical conundrum can easily confuse the clinician. Clinical astuteness and a high index of suspicion are paramount if successful management is to be instituted.

### Case presentation

We present the case of a 16-year-old Caucasian man who attended the acute admissions unit of our hospital with a history of an acutely painful, red, and swollen right hemiscrotum for about five to six hours. No history of testicular trauma was elicited. He also complained of vague, generalized abdominal pain, somewhat worse in the periumbilical region and lower abdomen, and vomiting and watery diarrhea for about 24 hours.

He had a temperature of 38.5°C and pulse rate of 95 per minute. On examination, his right testicle was tender and somewhat elevated, and the right hemiscrotum, red and swollen. Abdominal examination evinced mild tenderness in his epigastrium and central abdomen, and less so in the right iliac fossa and suprapubic area. No convincing localizing abdominal signs were noted. Blood tests showed a white cell count of 15 × 109/L, neutrophilia, and C-reactive protein of about 300 mg/L.

Clinically, testicular torsion could not be excluded, and, on account of his young age and scrotal signs, a decision was made to explore the scrotum. At surgery, on opening the right tunica vaginalis, approximately 5 ml of pus was found, apparently coming down from the right groin. His right testicle appeared entirely normal. The scrotal abscess was drained, the area washed thoroughly with saline, and the scrotal wall closed in two layers with absorbable sutures. A Lanz incision was made to explore his right iliac fossa. It was found that he had a perforated retrocecal appendix, resulting in an abscess extending into the pelvis. The appendix was excised and the abdominopelvic abscess drained. Thorough saline lavage was performed, and the wound was closed without insertion of an abdominal drain. He was given three days of postoperative intravenous antibiotics.

The patient made an excellent recovery and was well at clinic follow-up four weeks later.

## Discussion

The processus vaginalis is a developmental outpouching of the peritoneum that is present from around week 12 of gestation. In boys, it precedes the testis in its descent from the abdominal to the scrotal position and usually closes during the period from a few weeks before to a few weeks after birth. The portion around the testis remains as the tunica vaginalis. The processus vaginalis has been found to be patent in 80% to 95% of male newborns, the incidence then decreasing to 60% at one year, 40% at two years, and 15% to 37% thereafter [1,2]. A patent processus vaginalis can present as a hydrocele or congenital inguinal hernia.

**Figure 1:**
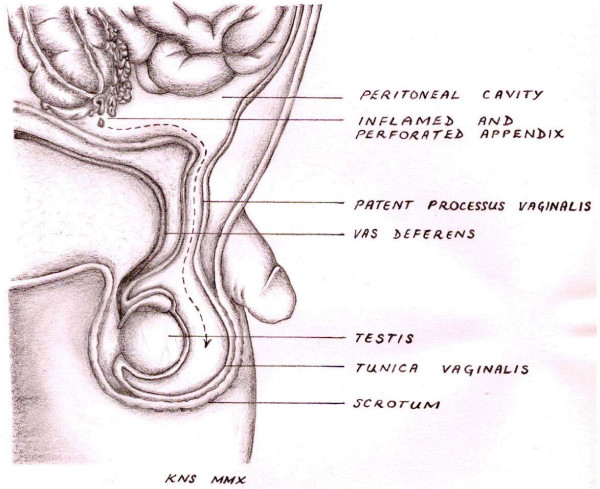
**Depiction of the path that pus from a perforated appendix follows *en route *to the scrotum**. This is possible only in the presence of a patent processus vaginalis.

Scrotal abscess as a complication of acute appendicitis is a very rare clinical entity. The condition occurs when pus from suppurative appendicitis tracks down through a patent processus vaginalis into the scrotum (Figure 1). To date, very few cases have been described in the literature. Scrotal abscess as a complication of appendectomy has been mentioned only very infrequently. A Medline search produced fewer than 20 reported cases [1,3-7]. A scrotal abscess occurring without a history of appendectomy, as happened in our patient, is even less common. A Medline search produced fewer than five such case reports [8-10].

The presentation of an acutely tender, red, and swollen testicle in a child inevitably raises the suspicion of testicular torsion. Surgical exploration in these cases should be done without delay if the correct diagnosis is to be corroborated and the viability of the testis ensured. Other possible causes of an acute scrotum include torsion of a testicular appendage, epididymo-orchitis, and incarcerated hernia.

As an intra-abdominal cause for acute scrotal signs is an extremely unusual clinical scenario, a good deal of discernment and lateral thinking is required to diagnose and manage the patient properly, as it can be tempting to explore the scrotum alone without having a look higher. Ultrasound imaging of the abdomen and scrotum can help in the diagnosis [10] but is no substitute for a high index of suspicion. Moreover, ultrasound may not be available at certain hours during an emergency surgical procedure.

## Conclusion

The possibility of an intra-abdominal abscess leading to the presentation of an acute scrotum secondary to a patent processus vaginalis should always be kept in mind. Thorough scrotal and abdominal lavage and removal of the source of sepsis are keys to successful management.

## Competing interests

The authors declare that they have no competing interests.

## Consent statement

Written informed consent was obtained from the patient's parents for publication of this case report and accompanying images. A copy of the written consent is available for review by the Editor-in-Chief of this journal.

## Authors' contributions

KNS managed the patient, wrote the manuscript, and drew the diagram. AAR managed the patient and made final changes to the manuscript. All authors read and approved the final manuscript.
